# Self-reported walking and associated factors in the Spanish population with chronic obstructive pulmonary disease

**DOI:** 10.1186/s12890-018-0731-4

**Published:** 2018-11-07

**Authors:** Pedro Barbolla Benito, Germán Peces-Barba Romero

**Affiliations:** 1 Autonomous University of Madrid, Ciudad Universitaria de Cantoblanco, Madrid, 28049 Spain; 2 Department of Pneumology IIS-Fundación Jiménez Díaz, Center for Biomedical Research in the Network, Respiratory Diseases (Spanish acronym CIBERES), Calle de Melchor Fernández Almagro, 3, Madrid, 28029 Spain

**Keywords:** Chronic obstructive pulmonary disease, Physical activity, Epidemiology, Spain

## Abstract

**Background:**

The level of physical activity among individuals with chronic obstructive pulmonary disease (COPD) is associated with the disease severity and prognosis. The aim of this study was to describe the prevalence of self-reported walking at least 150 min per week and the associated factors among the Spanish population with COPD.

**Methods:**

Analyses were based on data drawn from the 2009 European Health Interview Survey in Spain (2009 EHIS). Twenty-two thousand one hundred eighty-eight subjects participated in the survey (response rate of 96.5%). Participants were classified according to international physical activity recommendations. The prevalence of walking among participants with and without COPD (≥40 years old) was described. Univariate and multivariate logistic regression models were used to study the association of walking with socio-demographic and health outcome variables.

**Results:**

Of the participants with COPD, 55.0% reached the minimum walking recommendations compared to 59.9% of the general population. The level of walking physical activity of the participants with COPD differed according to sex, age, educational level, area of residence, living as a couple, self-rated health status, mental health, body mass index and hospital admissions. In the multivariate analysis, being male, < 65 years old, living in an area with ≥50,000 inhabitants, no diagnosed depression or anxiety and self-reported good to very good health were factors significantly associated with walking ≥150 min per week.

**Conclusions:**

Sex, age, area of residence, mental disorders and self-rated health are associated with weekly walking time in the Spanish population with COPD.

## Background

Chronic obstructive pulmonary disease (COPD) is a common, preventable and treatable disease characterised by persistent respiratory symptoms and limited airflow, generally associated with exposure to harmful agents such as tobacco. The most frequent symptoms are cough, dyspnea and sputum production [1]. The prevalence of this disease in Spain is about 10% of adults aged over 40 years old, being almost three-times higher in men [2]. There are also important geographical variations in the distribution of the COPD population, as well as the diagnosis and treatment [3].

Physical inactivity is one of the main risk factors for global mortality, and is associated with the development of multiple health problems and chronic diseases [4]. The American College of Sports Medicine (ACSM) recommends physical activity equivalent to at least 150 min of moderate activity a week for healthy adults as well as for people suffering from a chronic disease [5]. However, it is documented that people with COPD usually undertake insufficient levels of physical activity [6]. As physical activity is related to relevant disease determinants including severity and prognosis, low activity levels could have an important impact in these patients [7, 8]. In addition, health outcomes such as quality of life and mental health are also associated with the level of physical activity performed by this population [9–11].

Walking is the most common physical activity modality performed by the general population. Moreover, it is a low-risk and accessible activity, and is associated with multiple systemic and emotional benefits [12]. According to the ACSM, walking is classified as a moderate intensity activity and is suitable to achieve the minimum physical activity recommendations [5].

Recent Spanish research has described the prevalence of different levels of walking physical activity among populations with COPD. The authors have also observed that lower self-reported walking times are related to worse markers of disease severity in COPD, such as BODE index, dyspnea score, CAT score, Global Initiative for Chronic Obstructive Lung Disease (GOLD) classification or COPD exacerbations. [13]. However, to the best of our knowledge, no studies have focused on the association of walking recommendations with socio-demographic and health determinants in a representative sample of the Spanish population with COPD. The objective of this study was twofold: [1] to describe the prevalence of weekly walking recommendations in people with and without COPD in Spain; and [2] to study the association between walking recommendations with socio-demographic variables, self-rated health status and mental health in a representative sample of the Spanish population with COPD.

## Methods

This descriptive study was based on data drawn from the 2009 European Health Interview Survey (EHIS) of the Spanish population. The survey was carried out by the National Institute of Statistics (NIE). The 2009 EHIS was a face-to-face interview survey, conducted between April 2009 and March 2010.

The study was carried out in all Spanish provinces. The survey used a three-stage sampling approach with stratification of the first-stage units, which represented the census section. The second-stage units involved the main family dwellings. Finally, an adult aged 16 years or older was selected to be interviewed within each household. Finally 22,188 subjects participated in the survey. The final response rate was 96.5% of the theoretical sample (*n* = 23,004) to be representative of the Spanish population. Other details of the survey can be found on the website of the NIE [14]. As this research was carried out using a publicly available and anonymised database, it was not necessary to obtain ethical approval to carry out the study.

### Socio-demographic characteristics, body mass index and hospital admissions

The socio-demographic characteristics assessed were sex, age (40–64 years or ≥ 65 years), educational level (no studies completed, primary studies completed or secondary studies completed or over), living as a couple (yes or no) and the number of inhabitants in their area of residence (< 50,000 or ≥ 50,000 inhabitants). The body mass index (BMI) was calculated from the self-reported height and weight. Those participants with a BMI ≥30 kg.m^− 2^ were considered “obese”. Participants were classified according to their smoking status as either “smokers” or “non-smokers”. Hospital admissions were assessed based on the question “have you been admitted at least 1 night in the last 12 months”, to which participants answered “no” or “yes”.

### Walking physical activity

The level of physical activity was obtained from the total time spent undertaking walking activity per week. The EHIS used the International Physical Activity Questionnaire-Short Form (IPAQ-SF), which aims to measure the total physical activity (work-related, transport-related and health-enhancing physical activity). The participants answered the questions: “during the last 7 days, on how many days did you walk for at least 10 minutes at a time?”; and “How much time did you usually spend walking on one of those”. The subjects were classified according to the minimum physical activity recommendations as either < 150 min per week or ≥ 150 min per week. The participants were also classified in relation to their level of physical activity as either low (< 150 min per week), moderate (≥150 to 299 min per week) or high (≥300 min per week). In both cases, the participants were classified according to the physical activity guidelines [5].

### Chronic obstructive pulmonary disease and comorbidities

The respondent was considered to have COPD when he or she answered affirmatively to the question “has a doctor told you that you suffer from chronic bronchitis, emphysema or COPD”. The chronic comorbidities included in the study were asthma, coronary heart disease, myocardial infarction, arthritis, cancer, diabetes, stroke and liver dysfunction. The participants were classified as having “none”, “one” or “two or more” of these comorbidities.

### Self-rated health and mental health

Self-rated health (SRH) was assessed based on the question “how would you describe your health status in general”. The five possible answers, scored on a scale ranging from 1 “very good” to 5 “very bad”, were grouped into three categories: good to very good, regular, and bad to very bad. The presence of mental disorders was assessed based on the questions “has a doctor told you that you suffer from chronic depression” and “has a doctor told you that you suffer from suffer from chronic anxiety”. Those who answered affirmatively to one or both of these questions were considered to suffer from a mental illness.

### Statistical analysis

All data were weighted according to the EHIS sample design. The sample characteristics of participants with and without COPD were described as the weighted sample size and percentage (%). The same was done for participants with COPD according to the minimum recommendations for physical activity. The prevalence of individuals who met physical activity recommendations between the COPD and non-COPD groups was compared using the chi-square test and logistic regression tests, adjusted for sex and age group. The level of physical activity in the COPD group was described for all variables. Weighted bivariable and multivariable logistic regression models were used to estimate the association between the minimum walking recommendations (≥150 min per week) and the rest of the variables in participants with COPD. Firstly, bivariable logistic regression models were used to study the role of each variable. Secondly, a multivariable regression model was performed with all the variables whose role was identified to be statistically significant in the bivariable models. All statistical analyses were performed using the IBM SPSS statistical package (version 20; IBM Corp. Armonk, NY). A *p*-value of < 0.05 was considered statistically significant.

## Results

The total number of subjects aged 40 years and older included in the study was 13,199 (6956 females and 6243 males). The prevalence of self-reported COPD was 7.9% (95% CI [7.4, 8.3]). The prevalence of COPD among participants aged 40–64 years was 5.3% (95% CI [4.8, 5.8]) and 13.1% (95% CI [12.1, 14.1]) in those older than 65 years (*p* < 0.001).

Table 1 shows the distribution of participants with and without COPD according to their socio-demographic characteristics and health determinants. Participants with COPD had a significantly lower level of education, higher prevalence of comorbidities, obesity and mental illness, and worse SRH. There were also significant differences in sex, age, living as a couple, and hospital admissions.

**Table 1:** Subjects characteristics with and without COPD in the 2009 EHIS

Variable	COPD (N/%)	No COPD (N/%)	*p*
Sex			
Males	530 (51%)	5709 (47%)	0.012
Females	508 (49%)	6443 (53%)	
Age group			
40–64	467 (45%)	8366 (68.8%)	< 0.001
≥ 65	571 (55%)	3785 (31.2%)	
Living as a couple			
Yes	707 (68.2%)	8933 (73.6%)	< 0.001
Educational level			
No studies	90 (8.7%)	392 (3.2%)	< 0.001
Primary studies	326 (31.5%)	2086 (17.2%)	
Secondary studies	619 (59.8%)	9657 (79.6%)	
Area of residence			
< 50.000	512 (49.3%)	5725 (47.1%)	0.171
≥ 50.000	526 (50.7%)	6426 (52.9%)	
Smoking status			
Yes	227 (24.1%)	2954 (25.6%)	0.313
BMI (cat.)			
< 30 kg.m^−2^	658 (70.8%)	9153 (80.7%)	< 0.001
≥ 30 kg.m^−2^	271 (29.2%)	2190 (19.3%)	
Comorbidities			
None	265 (25.6%)	7909 (65.3%)	< 0.001
1	379 (36.7%)	2985 (24.6%)	
≥ 2	391 (37.8%)	1226 (10.1%)	
Self-rated health			
Very good/good	315 (30.4%)	7929 (65.2%)	< 0.001
Fair	401 (38.6%)	3016 (24.8%)	
Bad/Very bad	322 (31.0%)	1206 (9.9%)	
Mental illness			
Yes	278 (27.0%)	1555 (12.8%)	< 0.001
Hospital admission			
Yes	234 (22.6%)	1122 (9.2%)	< 0.001
OVERALL	1038 (7.9%)	12,151 (92.1%)	

Chi-square test statistical significance (*p*-value < 0.05)

**Fig. 1:**
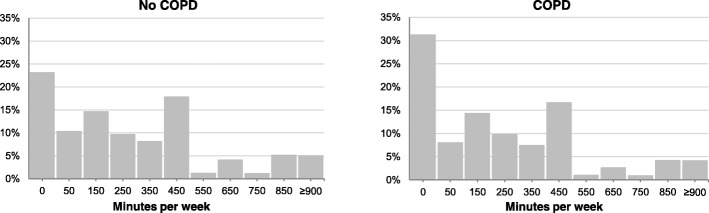
Distribution (%) of the participants with and without COPD according to the total walking time (minutes per week). Abbreviations: COPD, Chronic Obstructive Pulmonary Disease

Table 2 shows the proportion of participants with and without COPD that undertook walking according to the physical activity recommendations. The prevalence of individuals who met walking recommendations (≥150 min per week) in the COPD group was 55% (95% CI [51.9, 58.1]) compared to 59.9% (95% CI [59.0, 60.8]) in the group without COPD (*p* = 0.002 for the chi-square test and *p* = 0.011 for the adjusted logistic regression). The percentage of people with COPD who declared that they did not walk for at least 10 min in a row on any day of the week was 31.2% compared to 23.1% of the general population (Fig. 1).

**Table 2:** Prevalence of adherence to walking recommendations (≥ 150 min per week) in COPD and non-COPD

Variables	COPD	No COPD	*p*
	% (95% C.I.)	% (95% C.I.)	
Sex			
Males	60.3 (56.1–64.6)	63.3 (62.1–64.6)	0.058^a^
Females	49.4 (45.0–53.9)	56.9 (55.6–58.1)*	0.030^a^
Age group			
40–64	62.5 (58.0–67.0)	61.4 (60.3–62.4)	0.623^b^
≥ 65	49.0 (44.8–53.1)	56.7 (55.1–58.3)*	< 0.001^b^
OVERALL	55.0 (51.9–58.1)	59.9 (59.0–60.8)*	0.011^c^

*95% CI* 95% Confidence Interval; *Statistical significance (*p*-value < 0.05) for chi-square test; ^a^p for the logistic regression adjusted by age group; ^b^p for the logistic regression adjusted by sex; ^c^p for the logistic regression adjusted by age group and sex

**Fig. 2:**
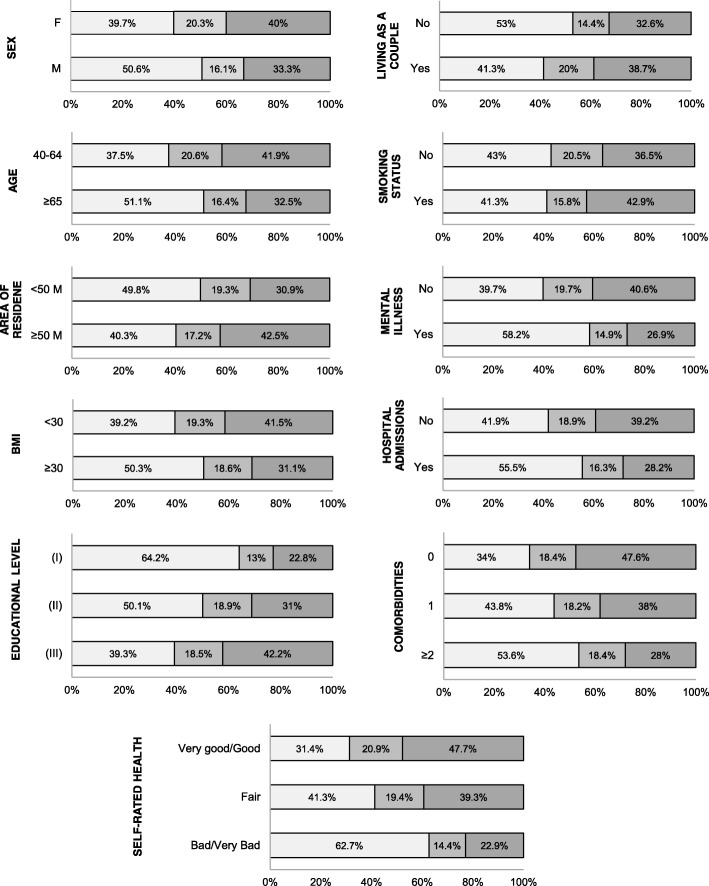
Percentage of people with COPD who report walking < 150 min per week (■), 150 to 299 min per week (■), ≥300 min per week (■) according to the sociodemographic variables, health status and metal health. Abbreviations: M, male; F, female; A. Residence: area of residence, M: thousand; (I), no studies; (II), primary studies completed; (III), secondary studies completed or over; BMI, Body Mass Index, < 30 kg.m^−2^ and ≥ 30 kg.m^− 2^

Table 3 shows the prevalence of self-reported walking (< 150 min per week or ≥ 150 min per week) in participants with COPD according to socio-demographic characteristics and health determinants. Figure 2 shows the level of physical activity (low, moderate and high level of walking activity) in participants with COPD according to the study variables. Those COPD participants aged 65 years and older, those with a lower educational level, those who did not live as a couple and those who lived in areas with < 50,000 inhabitants seem to be less active. In addition, a higher prevalence of low walking levels was observed among those suffering from another physical or mental disease, obese participants and those with worse SRH. People who had to be hospitalised at least once over the last 12 months for any reason also tended to be less active.

**Table 3:** Prevalence of self-reported walking (< 150 min per week or ≥ 150 min per week) in participants with COPD according to socio-demographic characteristics and health determinants

Variables	< 150 min/week (N/%)	≥150 min/week (N/%)	*p*
Sex			
Males	202 (44.8%)	308 (55.9%)	0.001
Females	249 (55.2%)	244 (44.1%)	
Age group			
40–64	167 (37.0%)	279 (50.6%)	< 0.001
≥ 65	284 (63.0%)	273 (49.4%)	
Living as a couple			
Yes	282 (62.4%)	401 (72.7%)	< 0.001
Educational level			
No studies	57 (12.7%)	32 (5.8%)	< 0.001
Primary studies	162 (35.9%)	161 (29.2%)	
Secondary studies	232 (51.5%)	358 (65.0%)	
Area of residence			
< 50.000	250 (55.4%)	252 (45.7%)	0.002
≥ 50.000	202 (44.6%)	300 (54.3%)	
Smoking status			
Yes	90 (23.2%)	128 (24.5%)	0.655
Comorbidities			
None	87 (19.3%)	169 (30.7%)	< 0.001
1	159 (35.2%)	203 (36.6%)	
≥ 2	205 (45.5%)	178 (32.3%)	
BMI (cat.)			
< 30 kg.m^−2^	251 (65.6%)	439 (74.9%)	0.002
≥ 30 kg.m^−2^	132 (34.4%)	112 (25.1%)	
Mental illness			
Yes	156 (35.1%)	130 (20.3%)	< 0.001
Self-rated health			
Very good/good	96 (43.7%)	209 (37.9%)	< 0.001
Fair	159 (35.1%)	225 (40.8%)	
Bad/Very bad	197 (43.7%)	117 (21.2%)	
Hospital admission			
Yes	127 (28.1%)	102 (18.4%)	< 0.001

Chi-square test statistical significance (*p*-value < 0.05)

Table 4 shows the association between walking and socio-demographic variables and health determinants in participants with COPD, analysed using bivariable and multivariable logistic regression models. In the bivariable analysis, walking ≥150 min per week was significantly associated with all study variables except for smoking status. In the multivariable analysis, sex, age, place of residence, mental illness and SRH were independently and significantly associated with minimum physical activity recommendations.

**Table 4:** Bivariable and multivariable logistic regression analysis for socio-demographic characteristics and health determinants in participants with COPD according to the walking recommendations (≥ 150 min per week)

Variables	≥ 150 min per week			
	Unajusted OR^a^ 95% CI	*p*	Ajusted OR^b^ 95% CI	*p*
Sex				
Females	1		1	
Males	1.56 (1.21–2.00)	< 0.05	1.49 (1.10–2.01)	< 0.05
Age group				
40–64	1		1	
≥ 65	0.58 (0.45–0.74)	< 0.05	0.69 (0.50–0.95)	< 0.05
Living as couple				
No	1		1	
Yes	1.61 (1.23–2.10)	< 0.05	1.18 (0.85–1.61)	NS
Educational level				
No studies	1		1	
Primary studies	1.78 (1.10–2.89)	< 0.05	1.79 (0.98–3.25)	NS
Secondary studies	2.77 (1.74–4.40)	< 0.05	1.72 (0.95–3.11)	NS
Area of residence				
< 50.000	1		1	
≥ 50.000	1.47 (1.15–1.89)	< 0.05	1.42 (1.07–1.88)	< 0.05
Smoking status				
No	1		–	–
Yes	1.07 (0.79–1.46)	NS	–	–
BMI (cat.)				
< 30 kg.m^−2^	1		1	
≥ 30 kg.m^−2^	0.64 (0.48–0.85)	< 0.05	0.75 (0.55–1.03)	NS
Comorbidities				
None	1		1	
1	0.66 (0.47–0.92)	< 0.05	0.89 (0.61–1.29)	NS
≥ 2	0.45 (0.32–0.62)	< 0.05	1.02 (0.67–1.54)	NS
Mental illness				
No	1		1	
Yes	0.47 (0.36–0.63)	< 0.05	0.68 (0.48–0.96)	< 0.05
Self-rated health				
Very good/good	1		1	
Fair	0.65 (0.47–0.89)	< 0.05	1.01 (0.70–1.47)	NS
Bad/Very bad	0.27 (0.20–0.38)	< 0.05	0.49 (0.32–0.75)	< 0.05
Hospital admission				
No	1		1	
Yes	0.58 (0.43–0.78)	< 0.05	0.76 (0.54–1.07)	NS

*OR* Odds Ratio, *95% CI* Confidence Interval, *NS* No Significant; ^a^unadjusted OR: bivariable analysis; ^b^adjusted OR: multivariable model adjusted for all variables with a statistically significant role in the univariate analysis; Statistical significance (*p*-value < 0.05)

## Discussion

In this study, being ≥65 years old was associated with less walking in people with COPD. The level of walking physical activity is associated with socio-demographic characteristics and the health status of people with COPD. In addition, the factors independently associated with reaching the minimum recommendations of physical activity (≥150 min walking per week) were sex, age, place of residence, better SRH and a lower prevalence of mental illnesses such as depression or anxiety.

In the present study, the differences observed in the level of physical activity among participants with and without COPD were rather low when compared to previous studies [15]. Two possible explanations are: firstly, participants of our study were drawn from a general representative sample of the Spanish adult population, therefore, they were not necessarily healthy, and secondly, we have only studied walking and we have not included other modalities of physical activity. Our results show that 55% of participants with COPD reported walking ≥150 min per week. This percentage is similar to that reported in previous studies in which 52% of patients with COPD met walking physical activity recommendations. [16], although somewhat higher than that obtained by Pitta et al. who observed that only 30% of patients with COPD reach ACSM walking recommendations [6]. However, two recent multicentre studies conducted in different Spanish populations with COPD have reported that the prevalence of low level of PA, defined as a walking time of > 30 min/day, was observed in a relatively low number of participants (86 and 85%, respectively) [11, 13]. These differences may be due to characteristics of the participants with COPD, the lack of physician confirmed diagnosis of COPD in our study and the location of the sample.

In our study population, the socio-demographic factors that were independently associated with physical activity recommendations were sex, age and the number of inhabitants in their area of residence. The association between the level of physical activity with age and sex is consistent with the literature. Younger adult population, particularly males, were more likely to achieve higher levels of physical activity [17]. However, in the INSEPOC study in Spain, no significant differences were observed in the total daily walking time between men and women with COPD [18]. Sex differences in self-reporting walking in our study should be interpreted carefully since the prevalence of COPD in female in the EHIS is usually higher than the prevalence reported by national and multi-centre epidemiological studies [2, 13, 15, 18].

Previous studies have shown that both younger and adult populations living in larger cities tend to be more physically active [19, 20]. Moreover, people living in smaller towns declare more environmental barriers, such us the lack of sidewalks, street lights, and difficulty in accessing facilities [19], which has been shown to play an important role in the level of physical activity of the population [21]. One study carried out in the city of Barcelona observed that two-thirds of daily walking trips exceeded 10 min [22]. These findings could help explain the results observed in the present study. Nevertheless, we were unable to find enough relevant studies in the literature to compare to our results. Our findings and the lack of information in the current literature highlight the need for future studies focusing on the role of environmental, geographic and social barriers in the level of physical activity in people with COPD.

Regular walking for at least 10 min every day in the COPD population could be a critical factor associated with mental and cognitive health status [23]. A recent study in Spain observed a clear relationship between walking less than 30 min a day and depression and a poorer quality of life in a group of patients with COPD. These researchers considered both quality of life and depression, which are both potential independent predictors of the level of physical activity of people suffering from this disease [11]. In the present study, participants who reached the minimum weekly recommendations for walking physical activity declared a better self-rated health status and lower prevalence of depression and anxiety. It has also been observed that the longer a person with COPD walks per day the lower the probability of suffering from depression [24]. However, it should be noted that our study did not take into account several relevant clinical determinants such as the BODE index, which has been shown to be closely related to depression in people with COPD [25].

We also observed that people who were admitted to hospital at least once during the past year for any reason tended to be less active. In addition, the bivariable analysis showed that hospital admission is inversely associated with self-reported walking recommendations. However, in the multivariable analysis this association was not statistically significant. Even so, previous epidemiological studies have shown a clear association between any type of regular physical activity and a lower risk of hospital admission due to exacerbated illness in this group of patients [8, 26].

### Limitations and strengths

Our results need to be interpreted within the context of the study’s limitations. The main limitation of the present study is related to its cross-sectional design, which prevented us from establishing any causal relationship. Another limitation is related to the way the information was obtained. The socio-demographic characteristics and factors associated with COPD were self-reported and, therefore, subject to bias and erroneous classification. However, this is compensated for by the high number of participants that are included in this type of health survey, which are periodically carried out. Moreover, the diagnosis of COPD was based on unvalidated self-reports, without having taken into account the results of a pulmonary function test, which may represent a bias related to the true prevalence of COPD within the population. Relevant clinical determinants of the disease, such as the BODE index, exacerbations, dyspnea or GOLD classification, which have been shown to be associated with the level of physical activity, could not be obtained from the 2009 EHIS [13]. However, this format has been previously used by other authors to study several aspects related to this disease in Spain, and reasonable reliability has been previously observed [27, 28].

Finally, the survey used the International Physical Activity Questionnaire-Short Form (IPAQ-SF) to assess physical activity, which could incorrectly estimate the real level of physical activity [29]. Moreover, some authors have demonstrated that the intensity of walking in in patients with moderate-to-very severe COPD does not reach the level of moderate physical activity [30]. It is well known that physical activity assessed from self-reported data is not as accurate as other objective assessment tools such as pedometers or accelerometers [31]. In addition, this questionnaire assessed physical activity as both walking for transportation and for leisure. Physical activity as leisure time has a positive association with perceived quality of life, while walking for transportation is inversely associated with quality of life [32]. Nevertheless, its validity and reliability are acceptable for studies with very large samples because objective measurements are not easy to take in representative surveys at a national level due to the high economic cost [33].

The strengths of this study include the sample size and country representativeness, meaning that the results can be generalised for the entire adult Spanish population with the same characteristics.

## Conclusions

To sum up, this study provides additional evidence that people with COPD undertake less self-reported walking activity than the general population in Spain, mainly in older participants. In addition, socio-demographic and health outcomes such as sex, age, area of residence, mental illness and self-rated health status are associated with self-reported weekly walking time recommendations in COPD participants.

Future large, prospective, population-wide studies are required to further explore the temporality of the associations observed between walking and socio-demographic and health outcomes in this investigation.

## Abbreviations

ACSM: American College of Sport Medicine
BMI: body mass index
CI: Confidence Interval
COPD: chronic obstructive pulmonary disease
EHIS: European Health Interview Survey
IPAQ-SF: International Physical Activity Questionnaire – Short Form
NIE: National Institute of Statistics
OR: Odds Ratio
SRH: self-rated health
